# New intrinsic mechanism on gum-like superelasticity of multifunctional alloys

**DOI:** 10.1038/srep02156

**Published:** 2013-07-08

**Authors:** Jia-Peng Liu, Yan-Dong Wang, Yu-Lin Hao, Yunzhi Wang, Zhi-Hua Nie, Dong Wang, Yang Ren, Zhao-Ping Lu, Jinguo Wang, Haoliang Wang, Xidong Hui, Ning Lu, Moon J. Kim, Rui Yang

**Affiliations:** 1 State Key Laboratory for Advanced Metals and Materials, University of Science and Technology, Beijing 100083, P. R. China; 2 School of Materials Science and Engineering, Beijing Institute of Technology, Beijing 100081, P. R. China; 3 Shenyang National Laboratory for Materials Science, Institute of Metal Research, Chinese Academy of Sciences, Shenyang 110016, P. R. China; 4 Department of Materials Science and Engineering, The Ohio State University, Columbus, Ohio 43210, USA; 5 Frontier Institute of Science and Technology, State Key Laboratory for Mechanical Behavior of Materials, Xi'an Jiaotong University, Xi'an 710049, China; 6 X-ray Science Division, Argonne National Laboratory, Argonne, IL 60439, USA; 7 Department of Materials Science and Engineering, The University of Texas at Dallas, Richardson, TX 75080, USA

## Abstract

Ti-Nb-based Gum Metals exhibit extraordinary superelasticity with ultralow elastic modulus, superior strength and ductility, and a peculiar dislocation-free deformation behavior, most of which challenge existing theories of crystal strength. Additionally, this kind of alloys actually displays even more anomalous mechanical properties, such as the non-linear superelastic behavior, accompanied by a pronounced tension-to-compression asymmetry, and large ductility with a low Poisson's ratio. Two main contradictory arguments exist concerning the deformation mechanisms of those alloys, i.e., formation of reversible nanodisturbance and reversible martensitic transformation. Herein we used the in-situ synchrotron high-energy X-ray scattering technique to reveal the novel intrinsic physical origin of all anomalous mechanical properties of the Ti-24Nb-4Zr-8Sn-0.10O alloy, a typical gum-like metal. Our experiments provide direct evidence on two different kinds of interesting, stress-induced, reversible nanoscale martensitic transitions, i.e., the austenitic regions with B2 structure transform to α″ martensite and those with BCC structure transform to δ martensite.

The strong, but less stiffer, metallic materials that exhibit high strength and low elastic modulus have long challenged the intrinsic physical nature of metallic bonds. The crystal strength is determined by a critical shear stress to actuate the glide of crystallographic line-defects called dislocations, which is in proportion to the shear elastic modulus, μ, i.e., approximately reaching 1/30 of μ^1^. For metallic alloys with a Poisson's ratio, ν, varying within a narrow range from 0.25 to 0.35^2^, the Young's modulus, E, is in linear relationship with the shear modulus via a simple relation E = 2(1 + ν)μ. This physical metallurgical principle was broken by a recent discovery on a series of multicomponent body-centered-cubic (BCC) solid solutions based on Ti-Nb binary alloys termed as gum metals (GMs) that have extraordinary multifunctional properties such as ultralow elastic modulus, ultrahigh strength, and super-elasticity and excellent plasticity^3^. Experimental studies found no phase transformations, no high density of dislocations (even after 90% cold work), and no twinning, leading to a hypothesis of dislocation-free deformation via the growth of nanodisturbances^4^ to form giant faults^5^,^6^. The activation of such mechanism was attributed to the combination of low ideal shear strength, induced by instability of the parent β phase^7^, and high critical shear stress required by conventional dislocation glide^3^,^6^. However, this hypothesis has been challenged by the well-known conventional deformation mechanisms, such as dislocation glide^8^, deformation twinning^8^,^9^, and stress-induced martensitic transformation from β (BCC) austenite to α″ (orthorhombic) martensite^9^,^10^,^11^, with essential differences from the nanodisturbance hypothesis in which the so-called ‘non-crystallographic' partial dislocations with the Burgers vectors having non-quantized magnitude exist and play a crucial role. Therefore, the underlying deformation mechanism responsible for the peculiar properties of this new class of metallic materials still remains elusive.

Here we use synchrotron-based high-energy X-ray diffuse scattering (HE-XRDS), in combination with computer simulations, to explore *in-situ* the deformation behavior of a gum-type Ti-24Nb-4Zr-8Sn-0.10O (in weight percent) alloy (abbreviated as Ti2448 from its chemical composition)^12^. Besides the high penetration, low absorption, great access to the reciprocal space, and high reciprocal resolution, the HE-XRDS technique can trace *in-situ* small changes in crystal structure, size, and elastic strain state even for nanocrystalline domains with a small volume fraction during deformation and phase transformation in bulk samples^13^. It is established in our present investigations that the Ti2448 alloy consists of a nanoscale B2 clusters embedded in a BCC matrix that has a slightly different Nb content from that of the B2 regions. Furthermore, our experiments provide unambiguous evidence on the formation of two different kinds of stress-induced nano-scale martensites, α″ and δ, originating from the B2 nanoclusters and the BCC matrix, respectively. The nano-scale martensites consist of frustrated nanodomains of individual martensitic variants, which are distinctively different from the normal self-accomodating polytwinned martensitic plates. We believe that stress-induced reversible transformations of B2 to α″ and BCC to δ and switch of nanodomains of these martensites contribute to the anomalous mechanical behavior of the gum-like metals.

## Results

**Figure 1:**
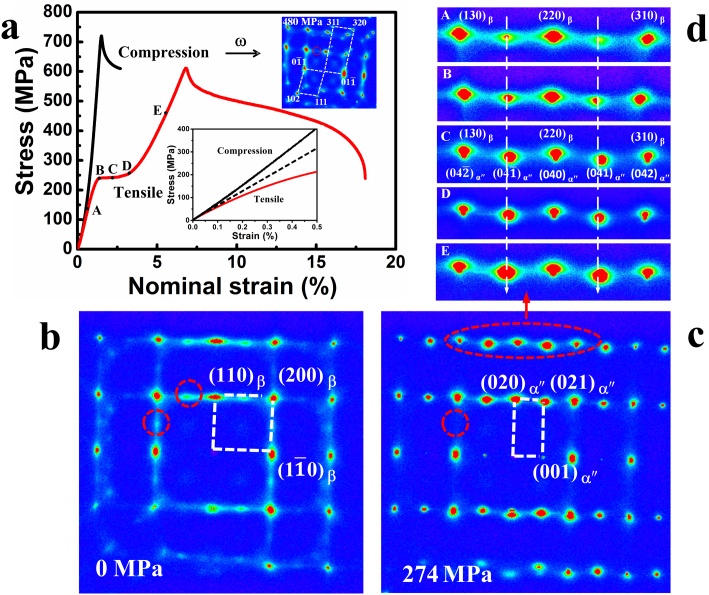
High-energy X-ray diffuse scattering (HE-XRDS) studies of deformation behaviour for gum-type Ti-24Nb-4Zr-8Sn-0.10O single crystal loaded along [110] orientation. (a), Stress-strain (SS) curve for single crystal under tensile and compression modes. (b), (c), The HE-XRDS pattern for the single crystal under tensile loading at 0 MPa (b) and 274 MPa (c) with the X-ray incident beam along [001]_β_ direction. Note that the existence of weak diffuse scattering signals located at the 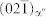 position is highlighted with red dotted circle in (b). (d), The detailed change in scattering patterns as highlighted in the red dotted circle in (c) at different stresses marked with the same symbols shown in (a). The insets in (a) are the scattering pattern under compression loading and enlarged S-S curve at a low strain, respectively, showing the diverse elastic modulus at different stress states. All newly-emerged diffraction spots appearing under compression mode can be indexed as ω phase.

Figure 1a gives the standard stress-strain (S-S) curves mechanically tested on single crystal Ti2448 loaded along the [110]_β_ direction under both tension and compression^12^ at room temperature. Interestingly, the two-step superelasticity with a stress plateau at ~240 MPa, accompanied by a jump in the strain of ~2%, is observed only under tension, in clear contrast to the almost linear S-S curve obtained under compression. Thus there is clearly a tension-compression asymmetry^12^. Moreover, the detailed changes in slopes of the S-S curves at low strain values (shown in the inset of Fig. 1a) indicate completely different non-linear elastic deformation behaviors under tension and compression. With increasing stress, an obvious reduction in modulus is observed under tension, but a slight increase in modulus is observed under compression.

The HE-XRDS reveals the existence of nano-sized domains of α" martensite^14^ prior to loading as evidenced from weak diffuse scattering signals at {021}_α″_ (indicated by the red circles) located at the middle of {110}_β_ and {200}_β_ diffraction spots (Fig. 1b). The nearly equal intensity of {021}_α″ _spots in the reciprocal space with cubic symmetry suggests no variant selection among the α" nano-sized martensitic domains. More importantly, the in-situ HE-XRDS experiments verify through distinctive changes in the scattering signals during tensile and compressive loading that *the superelastic behavior of the alloy involves complex phase transformation processes*. Under tension the stress-induced phase transformation from B2 nanoclusters to α" is evidenced by the obvious increase in the (021)_α″_ diffuse scattering signals (Fig. 1c), while the same phase transformation is suppressed under compression (as evidenced by the vanishing of the (021)_α″_ diffuse scattering signals) and, instead, ω phase starts to form at a compressive stress of ~500 MPa (as indicated by the HE-XRDS pattern displayed in the inset of Fig. 1a). After the applied compressive stress is removed, the ω phase remains (as indicated by the HE-XRDS patterns provided in the Supplementary Fig. S5 and Movie S1).

The crystal structure of α" is determined by using high-energy X-ray diffraction (HE-XRD) by rotating the sample around [110]_β_. It is a C222 orthorhombic structure (space group No. 21) with lattice parameters of *a* = 3.222 Å, *b* = 4.788 Å and *c* = 4.667 Å. It is *an ordered structure* and the corresponding disordered structure has a space group Cmcm (No. 63). Most of the diffraction spots of α" overlap with those of the β phase, indicating a good lattice match between α" and β at the given orientation relationship, i.e., (110)_β_//(010)_α″_ and [001]_β_//[100]_α″_. Some superlattice diffraction spots, such as (021)_α″_ and (041)_α″_, are from the α" phase due to both atomic occupation order and lattice modulation. The lattice modulation could be caused by the {110}_B2_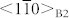 lattice shuffle involved in the stress-induced phase transformation from B2 to α" through the phonon softening mechanism. Due to an obvious difference in elastic modulus between α" and β as seen from their different diffraction elastic moduli calculated from the slop of their respective lattice strains (see the Suplimentary Information (SI)), even those fundamental overlapping lattice peaks finally separated under a tensile stress. The detailed phase transformation path during tensile loading can be seen clearly from the increase in the (041)_α″_ scattering signals at different stresses (Fig. 1d), as indicated by the different letters marked on the S-S curve in Fig. 1a. It should be noted that, with increasing tensile stress, the d-spacing of α" along the loading direction becomes larger than that of β as indicated by a rapid shift in the peak position belonging to (041)_α″_ scattering toward the beam center. Finally, the (220)_β_ diffraction peak splits into two peaks, i.e., (220)_β_ and (040)_α″_ (see the Supplementary Movies S2 and S3), which suggests that the α" phase is much less stiffer than the β phase. After the applied stress was removed, all the diffuse scattering spots returned to the initial state, suggesting that *the phase transformation is reversible* in contrast to the irreversible phase transformation under compression along [110]_β_.

**Figure 2:**
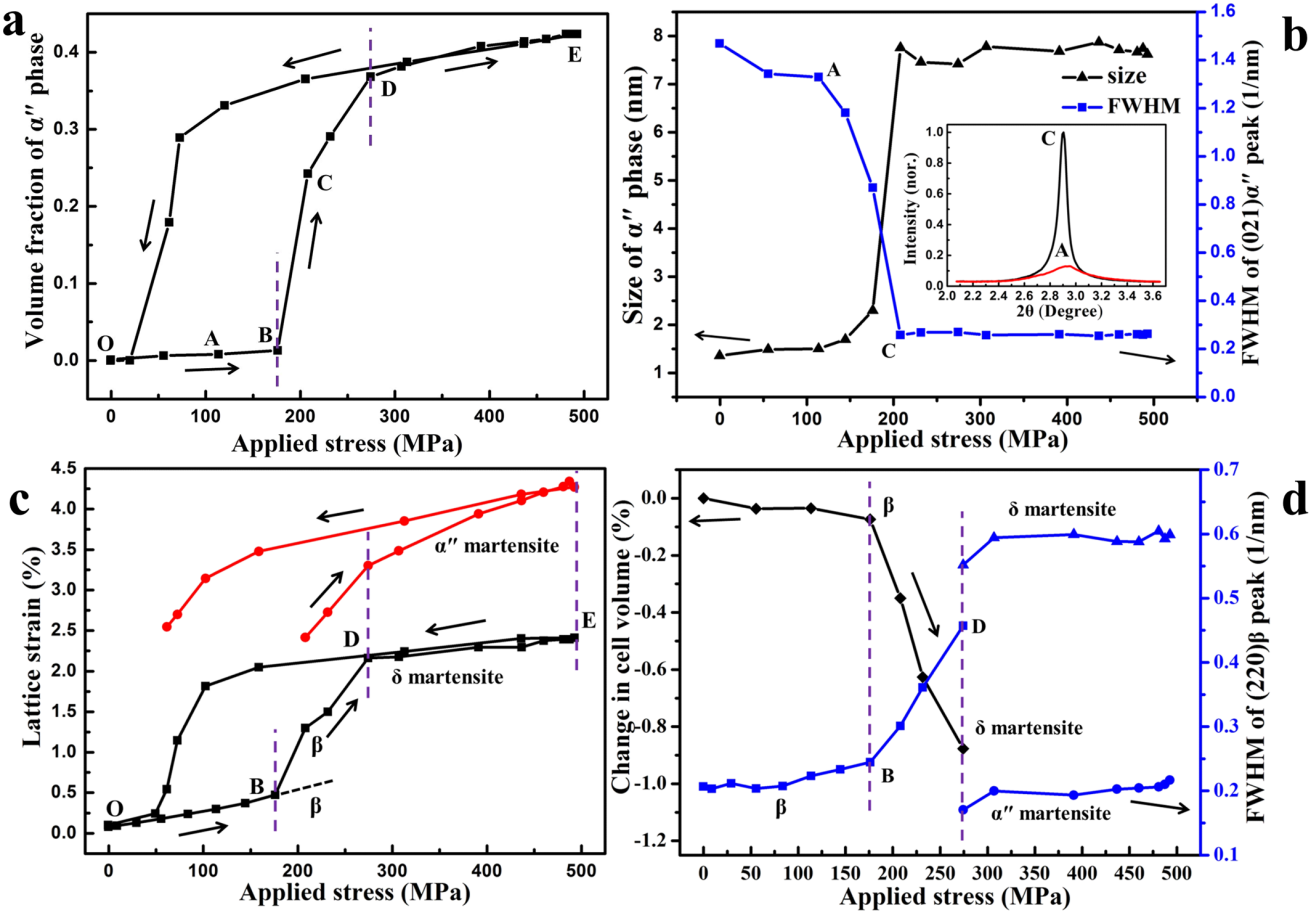
High-energy X-ray diffuse scattering (HE-XRDS) studies of phase transition kinetics and microstructural features for different phases for gum-type Ti-24Nb-4Zr-8Sn-0.10O single crystal. (a), Volume fraction of ordered α" phase as a function of applied stress. (b), The domain size and full-width at half maximum (FWHM) for 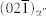 scattering peak of frustrated α" martensite as a function of applied stress. (c), The change in lattice strain obtained from (220)_β_ for β phase and frustrated δ martensite, and (040)_α″_ for α″ martensite as a function of applied stress. (d), The FWHM for β, δ, and α" phases as a function of applied stress. The inset in (b) is the scattering patterns of 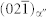 at different transformation stages. Note the change of cell volume from β to frustrated δ martensite under a tensile loading stress in (d).

The detailed analyses of the HE-XRDS data offer rich information on the phase transformation kinetics (PTK), the change in domain size (DS), and the local lattice strain of martensitic domains, for perceiving the microscopic deformation mechanisms in this interesting alloy system. Fig. 2a shows the variation of volume fraction (VF) of α" as a function of applied stress, σ, which is obtained from the change in the diffuse scattering integral intensity for its (021)_α″_ peak using a proper scale method (described in the SI). Fig. 2b shows the corresponding change in DS of α" derived from the full-width at half maximum (FWHM), as demonstrated in the inset of Fig. 2b. Based on the PTK for α", three different transformation regions are defined: (I) a slight increase in VF, accompanied by a slight increase in DS (from ~1.5 to ~2.2 nm) (noted as O-A-B); (II) a quick increase in VF, accompanied by an initially rapid increase in DS from ~2.2 to ~8 nm that remained constant thereafter (noted as B-C-D); and (III) a slow increase in VF with a constant DS (noted as D-E). It should be noted that there is a slight shift in the stress level between the macroscopic S-S curve (Fig. 1a) and the PTK curve (Fig. 2), which is due mainly to the different strain rates used in the two studies (see supplementary information). The most pronounced change occurred in the transformation regions from B–D.

The almost constant value in FWHM for the (021)_α″_ peak (shown in Fig. 2b) after entering into the transformation region (C-D-E) provides a convenient way to separate the lattice strain and FWHM for α" and β from the overlapped diffraction peaks (as shown respectively in Figs. 2c and 2d). The slope of the lattice strain vs. applied stress curve remains almost constant at low stresses (O-B in Fig. 2a), from which a diffraction elastic modulus of ~58 GPa was estimated in the initial stage of elastic deformation and ~30 GPa when the applied stress reaches ~180 MPa. Those values are in good agreement with the average Young's modulus measured by the mechanical method.

The lattice expansion caused by the phase transformation from β to α" can be evidenced from the sudden jump in the lattice strain curve of α" (Fig. 2c), which should not be surprising due to completely different crystal structures of α" and β. The astonishing finding is the existence of a sudden jump in both lattice strain and FWHM for β, corresponding exactly to the transformation from β into α". This indicates that, besides the stress-induced α" phase, there is another specific phase transformation from β to a new phase. Our dedicated HE-XRD experiment verified that the new phase has an orthorhombic structure, with lattice parameters of *a* = 3.190 Å, *b* = 4.762 Å, and *c* = 4.680 Å. Although this structure is close to that of the α" phase with similar lattice parameters, it belongs to a different space group (with the symbol cmcm, No. 63) (see the detailed change of cell parameters under loading in the supplementary information). We refer to this new phase as δ hereafter. The large value of FWHM and abrupt decrease in the unit cell volume (~ −0.9%) as shown in Fig. 2d after entering the region D–E indicate that the remaining matrix has been transformed into nanodomains of individual variants of the δ martensite. The main difference between the δ-phase and the α"-phase is that the former is a disordered structure without lattice modulation, while the latter is an ordered structure with a shuffle of adjacent {110}_B2_ planes, which could originate from different regions (e.g., nano domains of B2 clusters embeded in the BCC matrix, with slightly different compositions as well, as will be discussed below) in the initially inhomogeneous microstructure formed during the sample fabrication. The large FWHM value obtained for the δ martensite indicates that its domain size is rather small. Based on the measured FWHM, the effective size, Dv = 3.4 nm, can be estimated from the Debye-Scherrer formula, Dv = λ/(H·cosθ) (where λ is the wavelength of the radiation and H is the integral breadth of a reflection located at 2θ)^15^. When the applied stress is further increased (D–E in Fig. 2a), both lattice strain and FWHM of the δ-phase remain almost constant.

**Figure 3:**
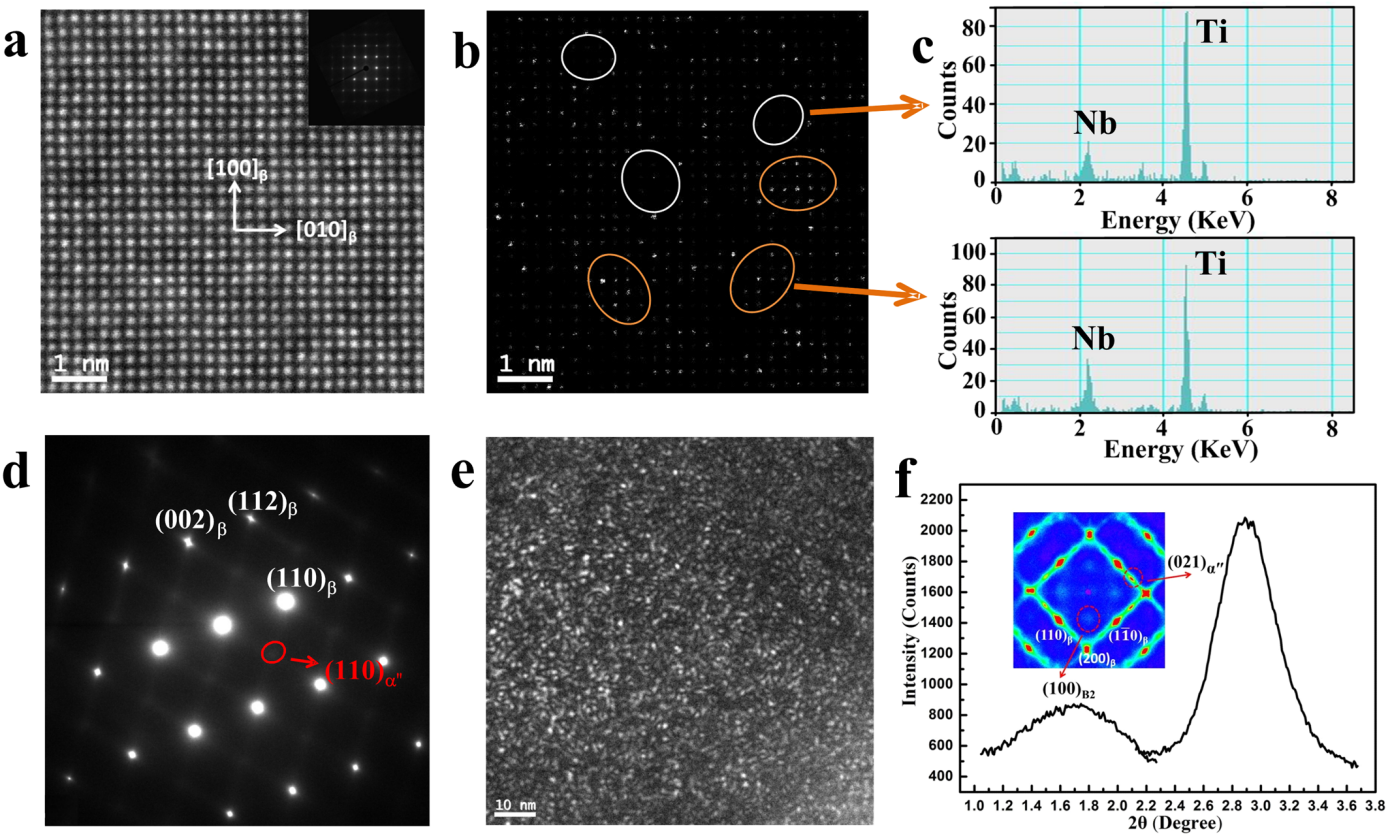
The TEM observations to show the origin of the different phase transitions. (a), (b), and (c) The HAADF-STEM image for [001]_β_ crystal axis with differently adjusted contrasts to show the existence of clusters of heavy atoms in the B2-ordered structure with the EDS spectra (c) at the Ti- and Nb- rich area marked inside those white and purple frames in (b). (d), (e), The nano-beam electron diffraction pattern to show the existence of weak diffuse scattering at 1/2(112)_β_ position and its corresponding dark field image to show the random distribution of α" nanodomains and B2 clusters inside the disordered BCC matrix. (f), The 1-dimensional HE-XRDS pattern to show the strong diffuse scattering signals located at (001)_β_ and (021)_α″ _positions.

Fig. 3a is the high-angle annular dark-field (HAADF)-scanning transmission electron microscopy (STEM) image with the incident electron beam along the [001]_β_ zone axis of the single crystal sample. The image (usually called as the Z-contrast image), formed only by very high-angle, incoherently-scattered electrons, as opposed to Bragg-scattered electrons, is highly sensitive to variations in the atomic number of elements contained in the sample. Heavier atoms show higher contrast, while lighter atoms appear darker in the image. The existence of heavy-atom (Nb, Zr, or Sn)-rich and -lean regions can be revealed by adjusting the contrast of the HAADF-STEM image (Fig. 3b). *The existence of two kinds of Nb-rich and Ti-rich phase-separated domains* is further confirmed by the spectra of energy-dispersive X-ray spectroscopy (EDS) (Fig. 3c), which show Nb-rich and Nb-lean regions. The existence of B2 clusters can be identified from the STEM image in the heavy-atom-rich areas (shown in Fig. 3b). The selected area electron diffraction pattern for the Ti2448 alloy along the [001]_β_ zone axis does not show an obvious diffuse scattering signal (see the inset of Fig. 3a), which is in contrast to that observed in the Ti-23Nb-1O alloy^16^. Different from what was reported in Ti-23Nb-1O alloy, our observation is due mainly to the small amount of oxygen and confirms that almost no modulated structure in B2 nanodomains was formed in the initial state before loading. The nano-beam electron diffraction pattern along the [011]_β_ zone axis indeed shows the existence of weak diffuse scattering located at 1/2(112)_β_ (in coincidence with (021)_α″_) position for Ti2448 (Fig. 3d), while its corresponding dark field image shows the random distribution of α" nanodomains and B2 clusters in the initial sample (Fig. 3e). The existence of the B2-clusters and α" nanodomains is supported strongly by the HE-XRDS signals located at the (001)_β_ (characterized with B2 structure) and (021)_α″_ peak positions for the undeformed Ti2448 alloy (shown in Fig. 3f).

## Discussion

**Figure 4:**
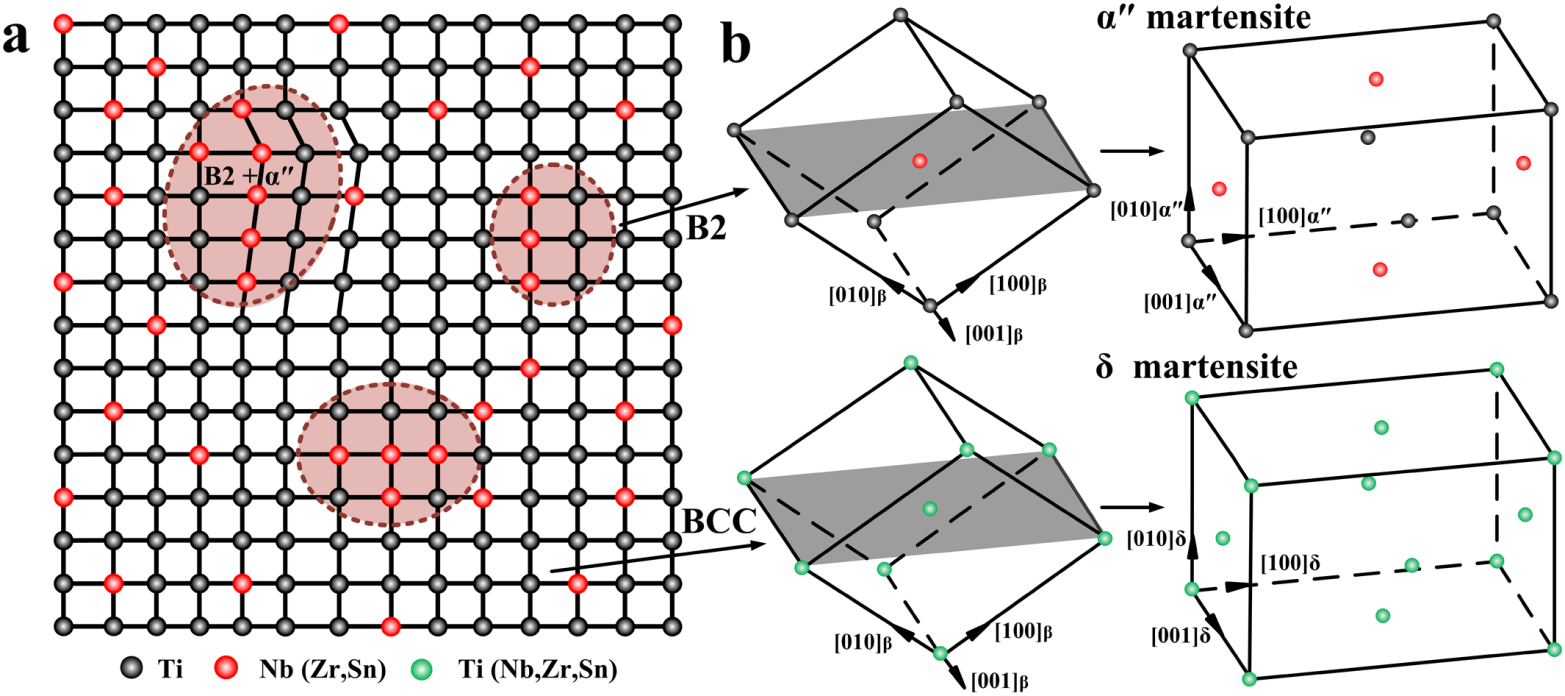
Schematics to show the dual martensite phase-transformation mechanism during deformation for gum-type Ti-24Nb-4Zr-8Sn-0.10O alloy. (a), The schematic diagram to show the initial microstructure that consists of three parts, i.e., Nb-rich B2 regions (austenite), Nb-rich α" martensitic domains (embryos) and Nb-lean BCC regions. (b), two kinds of phase transition scenarios during tensile deformation, i.e., the formation of (ordered) frustrated α"-martensite from B2-ordered structure and (disordered) frustrated δ-martensite from BCC-disordered structure.

The phase transformation described above for the Ti2448 alloy is completely different from that of normal martensitic transformation (NMT). Based on the TEM observations, the sample before loading is non-uniform in both structure and composition and consists of three regions: Nb-rich B2 clusters (austenite), Nb-rich α" static nano-scale domains (could also be called as embryos because they grow only under load and return to their original states when the load is removed) and Nb-lean BCC regions, as schematically shown in Fig. 4a. Upon loading, the existing α" embryos grow and the B2 and BCC regions transformed to nanodomains of α" and δ martensites, respectively, as schematically shown in Fig. 4b. Both the number and size of the nanodomains of these two kinds of martensites increase under the applied load, leading to the observed superelasticity and nonlinear elastic behavior of the system. But their growth is confined by chemical and structural non-uniformities in the austenite and by each other as neighboring domains. As no martensitic domains of any other variants are found in the whole HE-XRDS patterns, all domains belong to the same variant of martensite that is favored by the tensile load.

The stress-induced martensites in our sample are different from the polytwinned, long-range ordered strain state consisting of multiple self-accommodating variants as those observed during NMT. The long-range ordered strain state is suppressed by the random spatial distribution of various chemically and structurally different nanodomains in the alloy (see, e.g., Fig. 3e) that transform into different martenstic phases upon loading. Also note that the chemical and structural (degree of B2 ordering) non-uniformities present in the parent austenite phase, whose homogenization requires atomic diffusion, are preserved in the stress-induced martensitic phases.

The fine-scale chemical and structural non-uniformities (i.e., ordered B2 clusters in the disordered BCC matrix) in the austenite observed in the experiment could be produced by spinodal decomposition followed by B2 ordering in the Nb-rich domains^17^ or by the pseudo-spinodal decomposition mechanism^18^,^19^ in which thermal fluctuations bring concentrations of local regions beyond a critical concentration, c_0_, at which both the ordered and disordered phases have the same free energy, resulting in congruent ordering that is followed by phase separation. The presence of the α" embryos could be attributed to the random substitutional alloying elements in the alloy such as Nb, Zr and Sn whose atomic sizes are quite different from the host atoms (Ti). Both experimental and theoretical studies have shown that point defects could broaden martensitic transition temperature and reduce martensitic domain size^20^,^21^,^22^.

**Figure 5:**
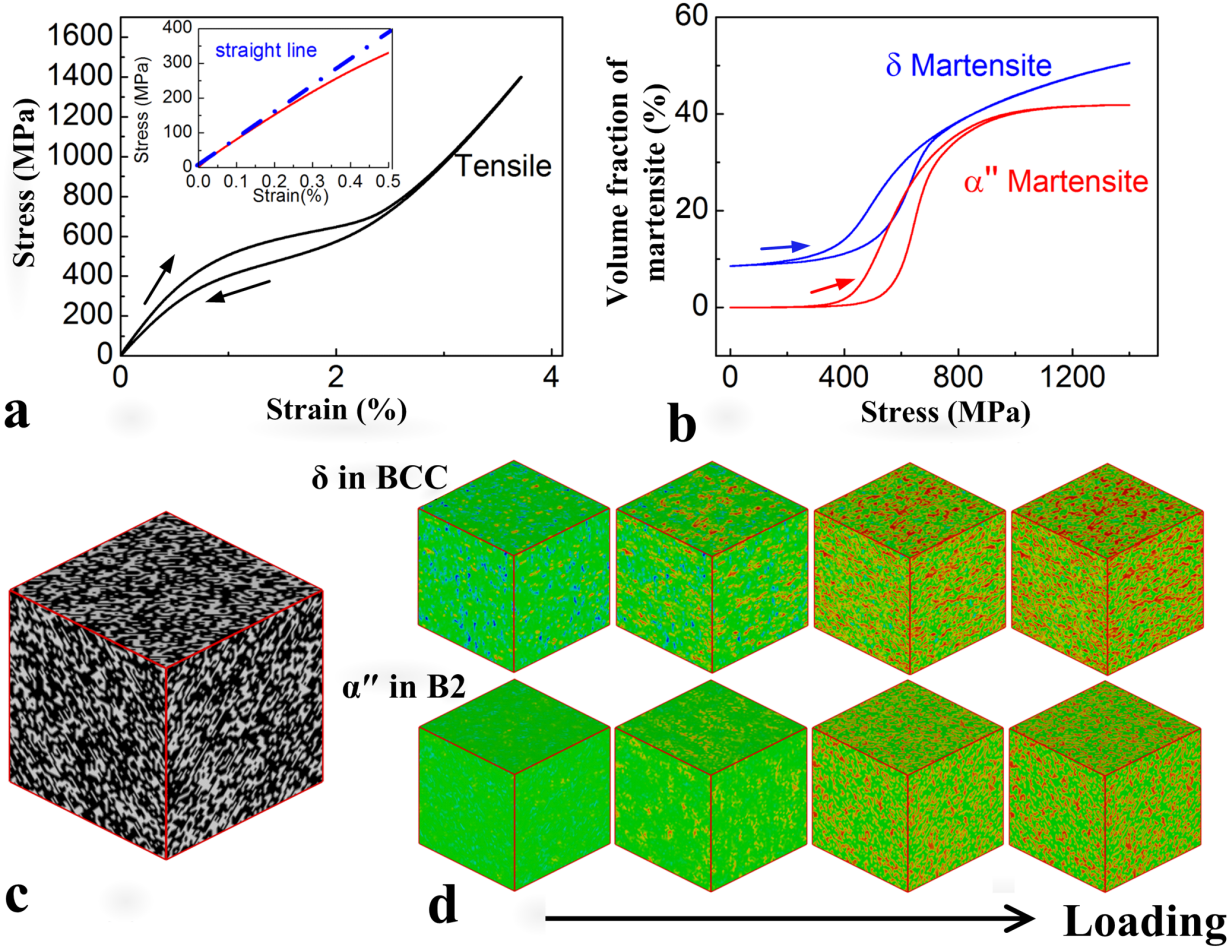
Phase field simulations on the deformation behaviour for gum-type Ti-24Nb-4Zr-8Sn-0.10O single crystal under tensile loading along [110] orientation. (a), Calculated tensile stress-strain curves along [110] direction, with the inset describing the enlarged S-S curves in elastic part. (b), Related martensitic volume fraction in disordered BCC regime and ordered B2 regime. (c), Ordered B2 structure (white color) and disordered BCC structure (black color) in our system. (d), Evolution of nano-sized martensitic domains in the ordered B2 regime and disordered BCC regime, respectively, under loading. There the green color describes the parent phase, red and blue colors describe the martensitic variants.

To understand the effect of these fine-scale chemical and structural non-uniformities in the austenite on MTs, we carry out computer simulations using the phase field method^21^,^23^,^24^ based on Landau theory. Note that our present simulations do not concern the kinetic processes that lead to the initial non-uniformities in the austenite. Instead we focus on the subsequent MTs under the influence of such non-uniformities in the parent phase. To do so we introduce two effects associated with these non-uniformities in our model, i.e., the local transition temperauture effect (LTTE) and local field effect (LFE))^21^,^22^. The LTTE allows us to have different stabilities (i.e., different martensitic start temperatures) for the two martensitic phases (i.e., α" in B2 regions and δ in BCC regions), while the LFE allows us to capture the effect of local lattice distortion caused by atomic size differences between the random substitional elements and the host atom mentioned above. In the model different LFEs are assumed for the BCC and B2 regions. Figure 5 shows the S-S curve and related properties of the Ti2448 alloy simulated by the phase field method. According to the experimental observations discussed above (Fig. 3b), the initial microstructure in our simulations (Fig. 5c) consists of Nb-rich (B2-structured) regions (light shade in Fig. 5c) with α" martensitic embryos and Nb-lean (BCC structure) regions (dark shades in Fig. 5c) with δ martensitic embryos. In our model, these martensitic embryos are induced by the random local fields associated with the substitutional elements. The B2 regions will transform into α" martensite while the BCC region will transform into the δ martensite.

Figure 5a shows the non-linear S-S curve under loading and unloading, with the inset showing the enlarged portion of S-S curve in the elastic region. Fig. 5b shows the calculated volume fraction change of martensitic domains in the BCC and B2 austenite matrix regions, respectively, as function of stress corresponding to the S-S curve shown in Fig. 5a. The volume fraction increases when the applied stress increases, resulting from the effect of stress-induced MT process. The stress-induced MT in both the BCC and B2 austenite occurs at a similar stress level (with different critical stress as shown in Fig. 5b). Fig. 5d shows the related microstructural evolution in the BCC and B2 austenitic regions, respectively, upon loading, where red and blue colors represent different martensitic variants (embryos of martensitic variants exist as shown by the light red or light blue regions) and green color represents the parent phase.

There are six martensitic variants for α" and δ respectively, and here we use the red color to describe the favorable martensitic variant under the applied stress and the blue color to describe the other unfavorable martensitic variants. When a load is applied, the system transforms gradually from a state consisting of nanodomains of all variants (red and blue in both BCC and B2 regions) at zero load into nanodomains dominated by one variant (i.e., the red one) that is preferred by the applied stress. The volume fraction curves (Fig. 5b) under loading also show the gradual increase of nanodomains of both α" and δ martensites. The gradual growth of existing domains, the newly-generated domains favored by the stress field, and the switch of unfavorable domains towards the favored ones upon the continued increase of the load are the intrinsic physical origins of both the non-linear stress-strain behavior in the elastic region and the pseudo-elastic behavior in the inelastic region of the stress-strain curve. Thus our computer simulations starting with nanodomains of BCC and B2 austinitic regions have shown the existence of two different stress-induced martensitic transformations and reproduced the deformation behavior of the Ti2448 alloy without considering any dislocation-related plastic deformations.

In sumary, the in-situ synchrotron-based high-energy X-ray scattering experiments, in combination with TEM observations reveal for the first time the existence of nanoscale chemical and structural non-uniformities and stress-induced dual martensitic transformations in Ti2448 GMs. The phase field simulations using the phase field method that takes into account of the nanoscale non-uniformities in austenite as well as possible effect from atomic size misfitting of the substitutional elements reproduce the deformation behavior of the alloy observed in the experiments. Thus the current study provides new insight into the intrinsic deformation mechanism for the anomalous mechanical behaviors of the gum-type metallic materials. The understanding of the physical origins of the stress-state dependence of elastic modulus during elastic deformation indeed benfits greatly designing new advanced functional and structural materials with wide applications in the whole materials spectrum.

## Methods

### Sample preparation

Master ingots with a composition of Ti-24Nb-4Zr-8Sn (mass%) were made by arc melting under argon protection and hot-forged at 1273 and 1123 K into bars 25 mm in diameter. Single crystals were grown from the as-forged bars using an optical floating-zone furnace (FZ-T-12000-X-VP-S, Crystal System Inc.) at a crystal growth rate of 5 mm h^−1^ under a high-purity argon gas flow. The seed crystals were cut along the <100>, <110> and <111> directions after their orientations were determined by both X-ray diffraction (XRD) and Laue X-ray back reflection analyses within 1 degree of the desired orientation and then used to grow single-crystal rods with the above orientations^12^. The dog-bone-shaped tensile and cube-shaped samples are cut from the grown single crystal for *in-situ* loading tension and compression tests, respectively.

### High-energy X-ray scattering and transmission electron microscope (TEM)

The *in-situ* X-ray diffuse scattering experiments were performed at the ID-11-C beam-line at the Advanced Photon Source (APS), Argonne National Laboratory (ANL). A monochromator with a Si (113) single crystal was used to provide an X-ray beam with the energy of 115 keV and a loading frame with a displacement control was applied to the beam-line for studying the change in crystallographic structure during tensile and compressive loading. The size of the X-ray beam was 0.5 × 0.5 mm. The transmission geometry in a manner similar to TEM was used to monitor the change in crystallographic structures during loading. A two-dimensional detector (Perkin Elmer amorphous silicon) was employed for collecting the scattering patterns over a plane, encompassing the transverse direction and (nearly) the loading direction of the single crystal, with an exposure time matching a simultaneous rocking of the specimen within ± 5° along the loading or transverse rotation axis for covering a large reciprocal space. Electron diffraction, dark field transmission electron microscopy (TEM), HR (high resolution)-TEM, HAADF-STEM and energy-dispersive X-ray (EDX) analyses were performed by using a JEOL ARM 200F microscope (JEOL, Tokyo, Japan) equipped with a Cs corrector, which was operated at 200 kV.

### Phase field simulation

The model system considered is a single crystal undergoing an austenite (BCC β phase) → martensite (orthorhombic phase) transformation^21^,^24^. The β phase is separated to 42% (volume fraction) Nb-rich ordered B2 structure and 58% Nb-lean BCC structure. B2 region and BCC region will transform respectively into α″ and δ martensitic phases, and the two martensitic phases have the same orthorhombic structure (See SI). To simplify the simulations, we assume that the B2 → α″ and BCC → δ transformations have the same transformation strain. We assume that the existence of alloying elements, such as Zr and Sn, in Ti2448 produces randomly distributed lattice distortion (i.e., local field effect) according to the atomic radius difference among Ti(2.00), Nb(2.08), Sn(1.72) and Zr(2.16).

## Author Contributions

Y.D.W., Y.L.H., Y.W., Z.P.L., R.Y. and Y.R. designed the research topic of this project. Y.L.H., H.W. and R.Y. prepared samples. Z.H.N., Y.R. and Y.D.W. performed synchrotron experiments. J.P.L., Y.D.W., Z.P.L. and X.H. analysed all synchrotron data. Y.W. and D.W. performed simulations. J.G.W., N.L. and M.J.K. performed T.E.M. study. Y.D.W., J.P.L., Y.W., Z.P.L., Y.L.H., D.W., Y.R., J.G.W. and R.Y. prepared the first version of manuscript and all authors took part in the discussions and paper writing.

## Supplementary Material

Supplementary Information Movie S1

Supplementary Information Movie S2

Supplementary Information Movie S3

Supplementary Information Supplementary Information for
