# Supplementary material for: New intrinsic mechanism on gum-like superelasticity of multifunctional alloys
Source: Sci Rep. 2013 Jul 8;3:2156. doi: 10.1038/srep02156 (PMC3703606; doi:10.1038/srep02156)
Supplement: Supplementary Information — for [file srep02156-s4.pdf]

Supplementary Information for “New intrinsic mechanism on gum-like superelasticity of multifunctional alloys”

Jia-Peng Liu, Yan-Dong Wang, Yu-Lin Hao, Yunzhi Wang, Zhi-Hua Nie, Dong Wang, Yang Ren, Zhao-Ping Lu, Jinguo Wang, Haoliang Wang, Xidong Hui, Ning Lu, Moon J. Kim and Rui Yang

In this part, we will provide some details on the high-energy X-ray diffuse scattering (HE-XRDS) method, scattering data analysis, crystallographic structure analysis, elastic modulus determination for different phases, and the phase field simulation methods with the input of materials parameters. The videos on the change in HE-XRDS during the tensile and compressive loading are also given to show the detailed scenarios of phase changes at different applied stresses and stress-state-dependent phase transformation.

A. High-energy X-ray diffuse scattering in-situ measurements

The HE-XRDS experimental setup is schematically shown in Fig. S1. A loading frame with a displacement control was applied at the synchrotron beam-line for studying *in-situ* the change in the scattering patterns to trace the evolution of crystallographic structure and microstructures under different loadings. The measurements of XRDS were performed at the exposure time that synchronized with continuous rocking along the X- and Y-axis. We performed the tensile loading and unloading for several times for one single crystal sample. The typical stress vs. displacement curve is displayed in Fig. S2 for one of single crystals under tensile loading and unloading along $[110]_\beta$ crystal axis. Note that the strain rate alters the stress-strain (S-S) curve, showing slight difference from the standard S-S curve (Fig. 1a) tested at a strain rate of 1.3×10^{-4} s^{-1}. There is an obvious difference in the stress plateau for the sample subjected to different loading cycles. The second-cycle loading displays a perfect superelastic recovery.

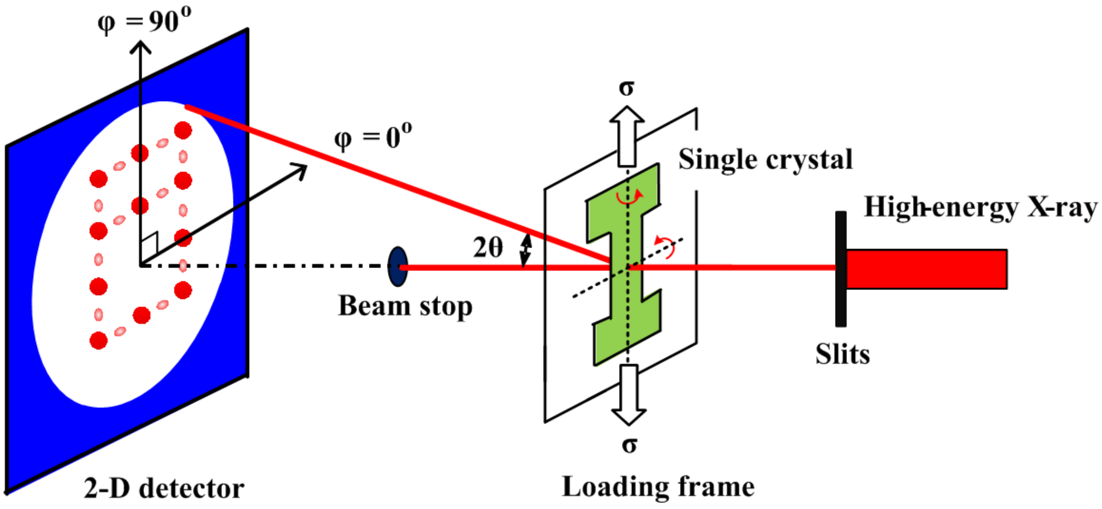

Figure S1 The schematic diagram to show the HE-XRDS experimental setup for tracing the evolution of crystallographic structures and martensite domains during in-situ loading.

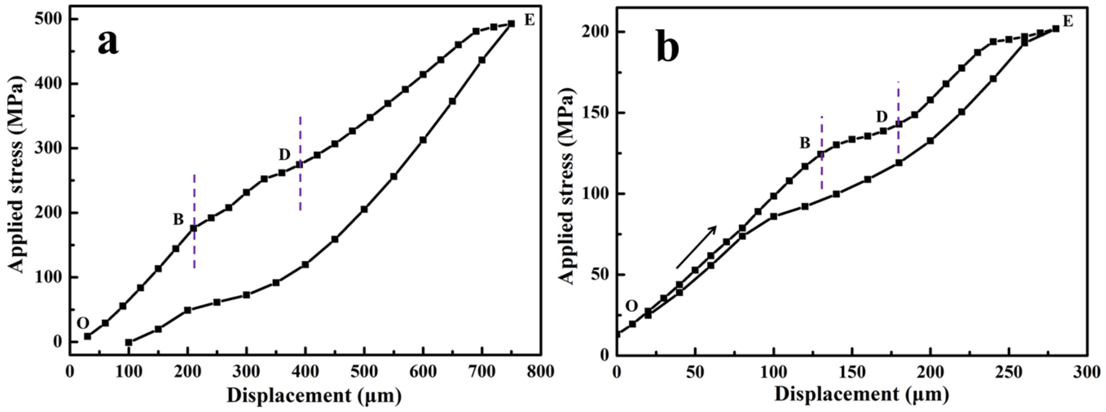

Figure S2 The stress vs. displacement curve for the Ti2448 single crystal under tensile loading along $[110]_\beta$ crystal axis: (a) the first time loading and unloading; (b) the second time loading and unloading.

B. X-ray scattering data analysis on the kinetics of phase transformation

Due to most of fundamental diffraction peaks for three phases, β, α″, and δ are overlapped, the kinetics of phase transformation under different loadings can only be traced through the superlattice diffraction peaks belong to α″. For example, the $(021)_{\alpha''}$

and $(001)_{\alpha''}$ peaks are typical ones used for determining the change in volume fraction of α″ under different loadings. Compared with those overlapped fundamental peaks, all superlattice peaks for α″ are very narrow even under a large applied stress, as shown in Fig. S3a for $(021)_{\alpha''}$ peak.

The change in both $(021)_{\alpha''}$ superlattice and $(220)_{\beta}/(040)_{\alpha''}$ overlapped diffraction spots were used to estimate the variation of volume fraction (VF) for α″ phase. On the one hand, the relative change in integral intensity for $(021)_{\alpha''}$ that belongs to the crystallographic lattice of α″ itself should be in proportion to VF of α″ phase. However, the absolute change in VF needs to be calibrated using the standard diffraction intensity by comparing the intensities for $(021)_{\alpha''}$ and $(220)_{\beta}$. However, due to lack of the information on the atomic ordering in the stress-induced α″ martensite, it is very difficult to compare the intensity of $(021)_{\alpha''}$ with that of $(220)_{\beta}$. It should be noted that previous Nb-lean β phase completely transforms into δ phase at a tensile stress of ~500 MPa and the overlapped diffraction peak for $(040)_{\delta}/(040)_{\alpha''}$ [or $(220)_{\beta}//(040)_{\alpha''}$] is well separated by fitting two Lorentz functions as shown in Fig. S3a. With this calibration method, the absolute variation of VF for α″ can be accurately obtained as a function of applied stress.

The well-separated $(040)_{\delta}$ and $(040)_{\alpha''}$ peaks, along with the $(021)_{\alpha''}$ single diffraction peak, are plotted on the same scale length ΔK of diffraction vector, as shown in Fig. S3b. There, ΔK is $2\Delta\theta\cos(\theta)/\lambda$ (θ is the diffraction angle and λ is wavelength). This plot gives a quantitative comparison of the contribution of the domain sizes to the diffraction peak width. It can be seen that the peak width for both $(040)_{\alpha''}$ and $(021)_{\alpha''}$ is very close, whereas that for $(040)_{\delta}$ is very broad. This indicates that the domain size for δ is very small.

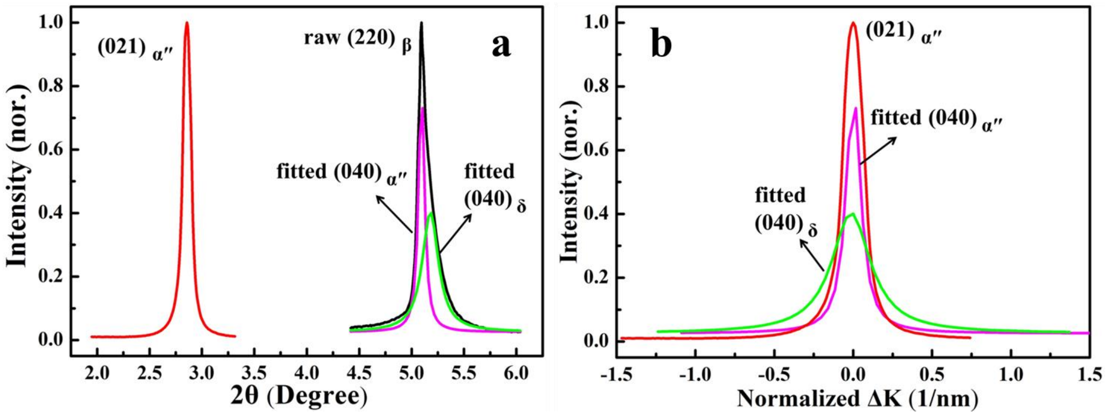

Figure S3 The one-dimensional diffraction profiles for (a) $(021)_{\alpha''}$ superlattice peak and $(040)_{\alpha''}/(040)_{\delta}$ overlapped peaks and (b) the normalized individual peaks for $(021)_{\alpha''}/(040)_{\alpha''}/(040)_{\delta}$ at the ΔK length scale.

C. Crystallographic structure determination

During the *in-situ* tensile loading along the $[110]_{\beta}$, the crystal is rotated around the loading direction (LD). The crystal structures of deformation-induced α″ and δ and their orientation relationship with β can be uniquely determined. Fig. S4 gives the indexed diffraction patterns for different phases before and after loading for the incident X-ray diffraction beam along the $[1\bar{1}0]$ crystal direction, i.e., the $[1\bar{1}0]$ crystal axis. The ordered α″ exhibits the obvious superlattice diffraction peaks as shown in Fig. S4b. Together with the [001] crystal axis shown in Fig. 1b and 1c, the orientation relationship between β and α″ (or δ) can be described as $(110)_{\beta}//(010)_{\alpha''}$ and $[001]_{\beta}//[100]_{\alpha''}$. Table S1 lists the detailed lattice parameters for the above-mentioned three phases. The changes in cell volume from β to α″ and β to δ are +0.29% and -0.91%, respectively.

During the *in-situ* compressive loading, the ω phase, rather than α″, was formed. Fig. S5 gives the indexed diffraction patterns for different phases before and after loading for the [001] crystal axis. After unloading, small amount of ω phase remained in the β matrix, which indicates that the phase transformation from β to ω is irreversible under compression. The orientation relationship between β and ω can be described as $(111)_{\beta}//(0001)_{\omega}$, $[01\bar{1}]_{\beta}//[11\bar{2}0]_{\omega}$.

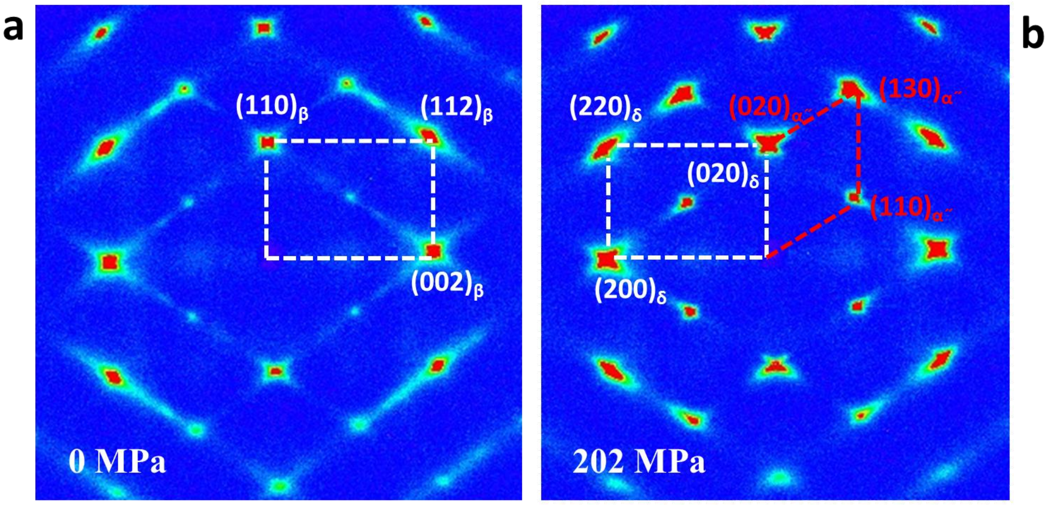

Figure S4 The HE-XRDS pattern for the single crystal under tensile loading at (a) 0 MPa and (b) 202 MPa with the X-ray incident beam along $[1\bar{1}0]_{\beta}$ direction.

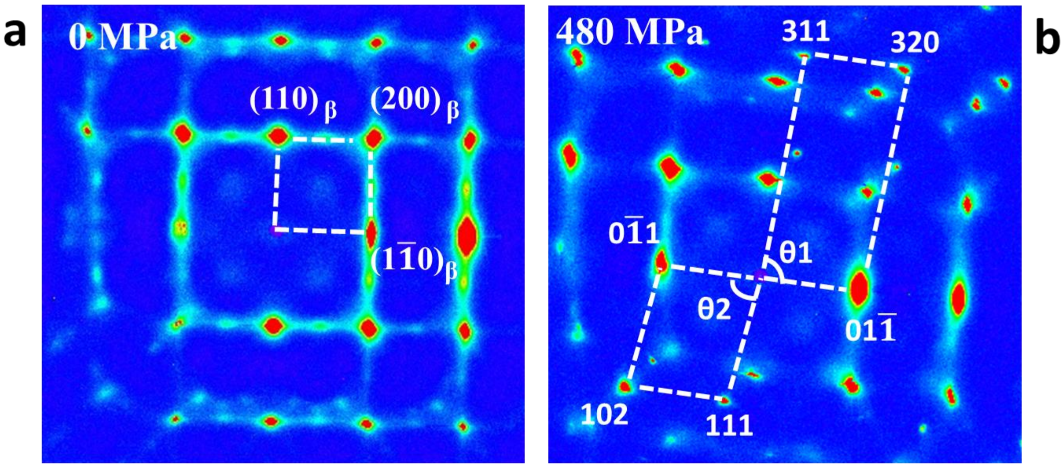

Figure S5 The HE-XRDS pattern for the single crystal under compressive loading at (a) 0 MPa and (b) 480 MPa with the X-ray incident beam along $[001]_{\beta}$ direction.

Table S1 The lattice parameters for three phases, β, α″, and δ determined by HE-XRDS

Phase	a (Å)	b (Å)	c (Å)
α″ martensite	3.222	4.788	4.667
δ martensite	3.190	4.762	4.680
β	3.298	3.298	3.298

D. Change in lattice parameters for δ phase

The detailed change in lattice parameters of two transverse planes, i.e., $(1\bar{1}0)_\beta$ and $(002)_\beta$, for the phase transformation from β to δ for the single crystal loading along $[110]_\beta$ is shown in Fig. S6. It can be seen that, with increasing applied stress, there is an obvious expansion in the transverse direction along the $[1\bar{1}0]$ orientation and a contraction along the [001] orientation. After entering into the BD stress stage, a sudden jump subjected to a small increased stress for the lattice strains along both transverse directions was evidenced. The alterations in the slope of lattice strain vs. stress for the transverse directions coincide well with the change along the loading direction (shown in Fig. 2c in main text), which provides further evidence for the phase transformation from β to δ. The present study explains also well the observed small value of Poisson's ratio in the studied alloy due to specified phase transformation.

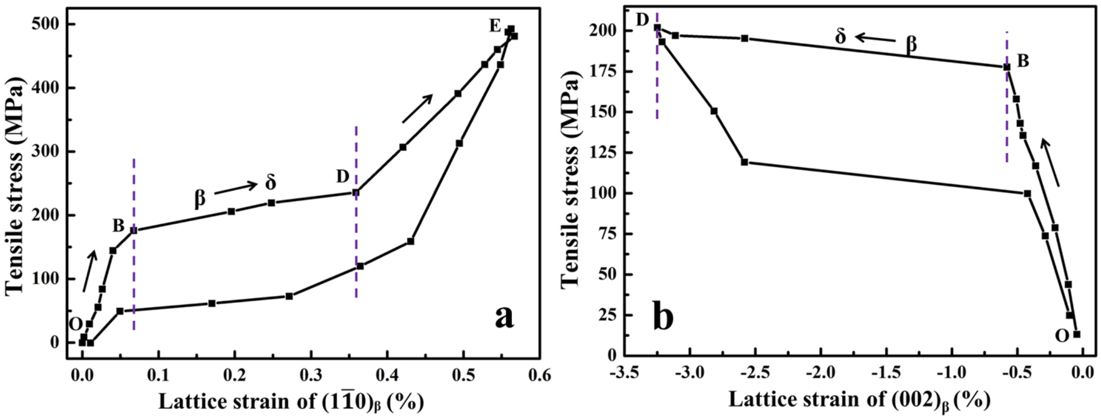

Figure S6 The change in lattice strain of (a) $(1\bar{1}0)_\beta$ and (b) $(002)_\beta$ as a function of applied tensile stress for the single crystal loading along [011].

E. The evolution of elastic modulus for β, α″, and δ phases

The diffraction elastic modulus for each phase was obtained by fitting the slope of the plots of their respective lattice strains vs. applied stress, on assumption of constant stress subjected to all phases. Due to the small domain size, this constant stress

assumption is reasonable. Due to the obvious non-linear relationship between the lattice strains and applied stress for β phase at the stress from 0 to 270 MPa (O-B-D stress region in Fig. 2c), we used separated second-order polynomials to fit. Fig. S7a shows that the diffraction modulus decreased from ~60 GPa to ~30 GPa when the applied stress was increased from 0 to 180 MPa (O-B stress region). While the applied stress was increased from 180 to 270 MPa (B-D stress region), the diffraction modulus changed from ~3.4 GPa to ~8.4 GPa, as shown in Fig. S7b, which indicates that the phase transformation occurred.

As shown in Fig. S7c and S7d, the linear-fitting was used for α″ and δ, respectively, after the phase transformation was completed. The diffraction modulus for α″ and δ are 21 and 84 GPa, respectively. The diffraction elastic modulus vs. applied stress curve for β, α″, and δ phases at different deformation stages are displayed in Fig. S8.

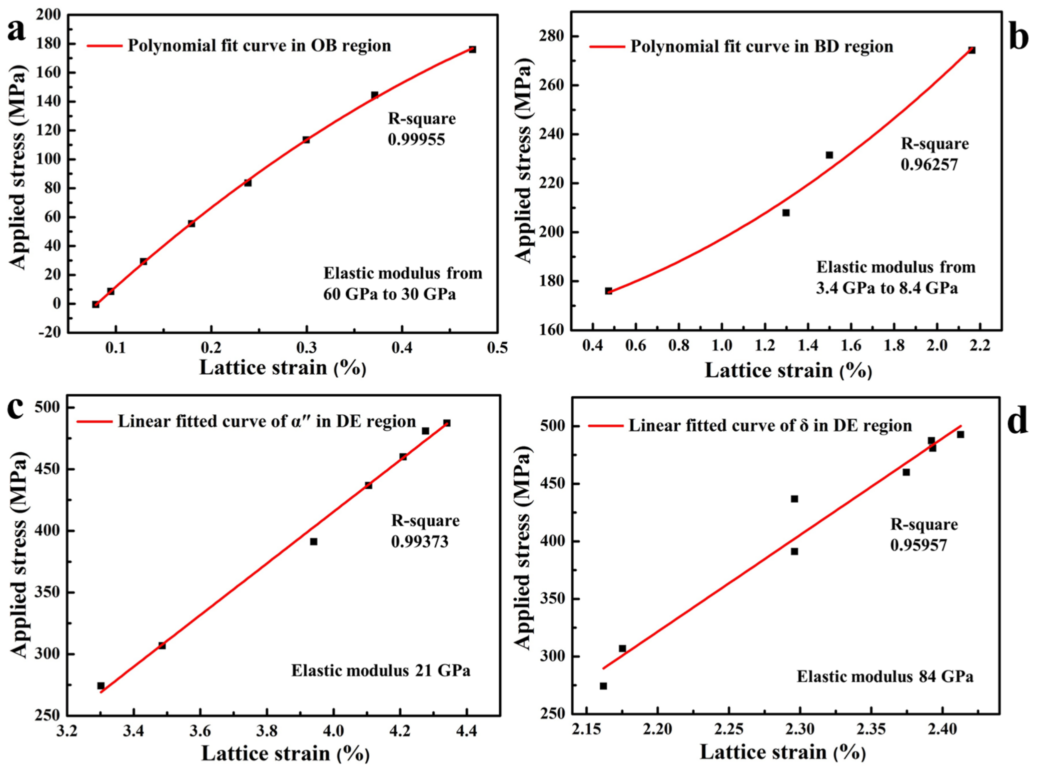

Figure S7 The slope of the lattice strain vs. applied stress curves to determine diffraction elastic modulus for β, α″, and δ phases in different deformation stages: (a) for β in the O-B deformation region, (b) for β in B-D deformation region, (c) for α″ at DE-deformation stage, and (d) for δ at DE-deformation stage.

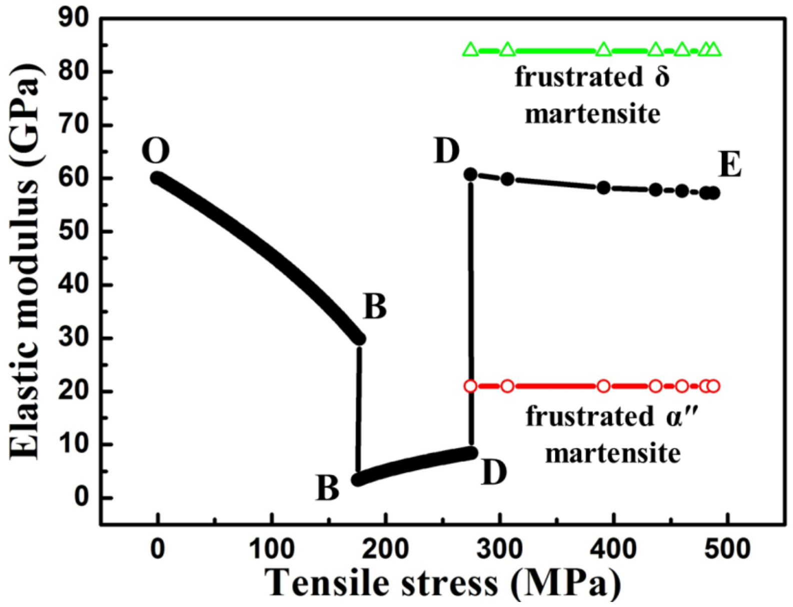

Figure S8 The diffraction elastic modulus vs. applied stress curve for β, α″, and δ phases at different deformation stages.

F. The phase field simulations on Ti2448 alloy during deformation

The model system was separated to two parts: 30% Nb-rich ordered B2 structure and 70% Nb-lean disordered BCC structure. Nb-rich ordered B2 part has less Zr or Sn than Nb-lean disordered BCC part, which results in different martensitic transformation temperatures. Recent precursory tweed/strain glass mechanism [1, 2] and stress-induced domain switching mechanism [3] are considered in our model. In our simulation, we assume B2→M and BCC→M have same transformation strain, so the total free energy, F, of the system can be formulated as

$$F = \int d^3\mathbf{r}[\, f_{ch}(\eta_1,\eta_2,\cdots,\eta_6) + f_L + f_{gr}(\eta_1,\eta_2,\cdots,\eta_6)] + E_{el} \tag{1}$$

which consists of four contributions [1, 2]: 1) local chemical free energy, $f_{ch}(\eta_1,\eta_2,\cdots,\eta_6)$, approximated by a Landau expansion polynomial with respect to the order parameters ηi, i=1-6 that characterize structural changes from A to M along the transformation pathway that preserves their lattice correspondence,

$$f_{ch}(\eta_1,\eta_2,\cdots,\eta_6)=\frac{1}{2}A_1\sum_{i=1-6}\eta(\mathbf{r})_i^2-\frac{1}{4}A_2\sum_{i=1-6}\eta(\mathbf{r})_i^4+\frac{1}{6}A_3(\sum_{i=1-6}\eta(\mathbf{r})_i^2)^3; \qquad (2)$$

2) excess free energy, f_L, caused by a local field effect (LFE) associated with the lattice distortions caused by the alloying elements (impurities); 3) non-local gradient energy terms, $f_{ch}(\eta_1,\eta_2,\cdots,\eta_6)$, that characterizes contributions to the total free energy from spatial non-uniformities in the order parameters; and 4) the coherent elastic strain energy, E_{el}, associated with lattice mismatch between the A and M phases and among different orientation variants of the M phase.

In Eq. (2) A1, A2 and A3 are the expansion coefficients and the leading term coefficient A1 depends on temperature and defect concentration. The chemical driving force for the A to M transformation is assumed to be $\Delta f_{A\to M}$ = -5.7×103 J/mole (i.e., 0.03 eV/atom) [4].

The variation of the stress-free transformation strain (SFTS) field in an arbitrary mixture of A and M phases can be described in terms of the order parameter fields [5, 6], i.e., $\varepsilon_{ij}=\sum_{p=1}^{3}\varepsilon_{ij}^{0}(p)\eta_p^2$, where ε_{ij}^0 is the stress-free transformation strain of α″ phase, which has been calculated through the lattice relationship between A and M in the Ti2448 system shown in Table S2.

$$\varepsilon^0(i)=\begin{pmatrix}0.0307 & -0.0321 & 0\\ -0.0321 & 0.0307 & 0\\ 0 & 0 & -0.0879\end{pmatrix}, i=1,2,\cdots,6.$$

Table S2 Lattice relationship between β and α″ phase

Orientation relationship	Equivalent d-spacing (β)	Equivalent d-spacing (α")
(001)β//(100)α"	3.298	3.222
(-110)β//(010)α"	2.334	2.394
(110)β//(001) α"	2.334	2.334

The excess energy (f_L) caused by the LFE is approximated by:

$$f_L(r) = \sum_{i,j,k,l=1,2,3} \varepsilon_{kl}^{local}(\mathbf{r}) \cdot \varepsilon_{ij}^{0}(\mathbf{r}). \tag{3}$$

where $\varepsilon_{kl}^{local}(\mathbf{r})$ describes local lattice distortions caused by the impurities and it depends on the defect's type and configuration. Here, a simple dilational distortion is assumed according to the atomic radius of Ti (2.0), Nb (2.08), Sn (1.72) and Zr (2.16). The atomic radius difference between Zr, Sn and Ti is larger than that between Nb and Ti, which results in Nb-lean disorderd BCC part possessing larger lattice distortion.

The non-local gradient term reads

$$\begin{aligned} f_{gr}(\eta_1,\eta_2,\cdots\eta_6) &= \frac{1}{2}\beta((\nabla\eta_1)^2+(\nabla\eta_2)^2+\cdots(\nabla\eta_6)^2) \\ &= \frac{1}{2}\beta(\sum_j^3\frac{\partial^2\eta_1}{\partial x_j^2}+\sum_j^3\frac{\partial^2\eta_2}{\partial x_j^2}+\cdots\sum_j^3\frac{\partial^2\eta_6}{\partial x_j^2}) \end{aligned} . \tag{4}$$

and the coherency elastic strain energy is formulated based on Khachaturyan's microelasticity theory:

$$\begin{aligned} E_{el} &= \frac{1}{2}c_{ijkl}\sum_{p=1}^{3}\sum_{q=1}^{3}\varepsilon_{ij}^{00}(p)\varepsilon_{kl}^{00}(q)\int\eta_p^2(\mathbf{r}\ )\eta_q^2(\mathbf{r})d^3r \\ &-\frac{1}{2}\sum_{p=1}^{3}\sum_{q=1}^{3}\int\frac{d^3k}{(2\pi)^3}B_{pq}(\frac{\vec{k}}{k})\{\eta_p^2(\mathbf{r})\}_k\{\eta_q^2(\mathbf{r})\}_k^* \end{aligned} . \tag{5}$$

The energy caused by applied stress field σ can be described as:

$$E_{applied} = -\sigma \cdot \bar{\varepsilon} = -\int \sigma_{ij} \cdot (\varepsilon_{ij}^{00}(1)\eta_1^2(r) + \varepsilon_{ij}^{00}(2)\eta_2^2(r) + \cdots \varepsilon_{ij}^{00}(6)\eta_6^2(r))d^3r \quad (6)$$

The stochastic time-dependent Ginsburg-Landau equation is used for the time-evolution of the order parameters during the simulation:

$$\frac{d\eta_p(\mathbf{r},t)}{dt} = -M\frac{\delta F}{\delta\eta_p(\mathbf{r},t)} + \zeta_p(\mathbf{r},t), \qquad p = 1, 2, \cdots 6\,. \quad (7)$$

where η_p is the order parameter of six martensitic variants, t is time, F is the total free energy, ζ is the noise term to describe the thermal fluctuation, M is the dynamic coefficient. The following parameters in dimensionless units are used in the simulations: The Landau expansion coefficients A_1^0 =0.02, A_2=32.05, A_3=37.5; dynamic coefficient M =1.0; elastic constants C_{11}=57.2, C_{12}=36.1, and C_{44}=35.9 [7]. The system size considered in the simulations is 64×64×64 and periodical boundary conditions are applied.

Figure S9a gives the phase field simulations on the S-S curve for gum-type Ti-24Nb-4Zr-8Sn-0.10O single crystal subjected to different tensile loading/unloading cycles along [110] orientation. It can be seen that a small residual strain exists at the first loading and unloading cycle, whereas there is almost no residual stress in the second cycle. The simulations agree with well the experimental S-S curve and the effect of loading cycles on the S-S curve as shown in Fig. S2. The related martensitic volume fraction in disordered B2 regime and ordered BCC regime under different loading cycles is given in Fig. S9b. It can be seen that the phase transformation kinetics on the martensitic volume fraction as a function of applied stress is almost stable after the first cycle loading and unloading for both phases.

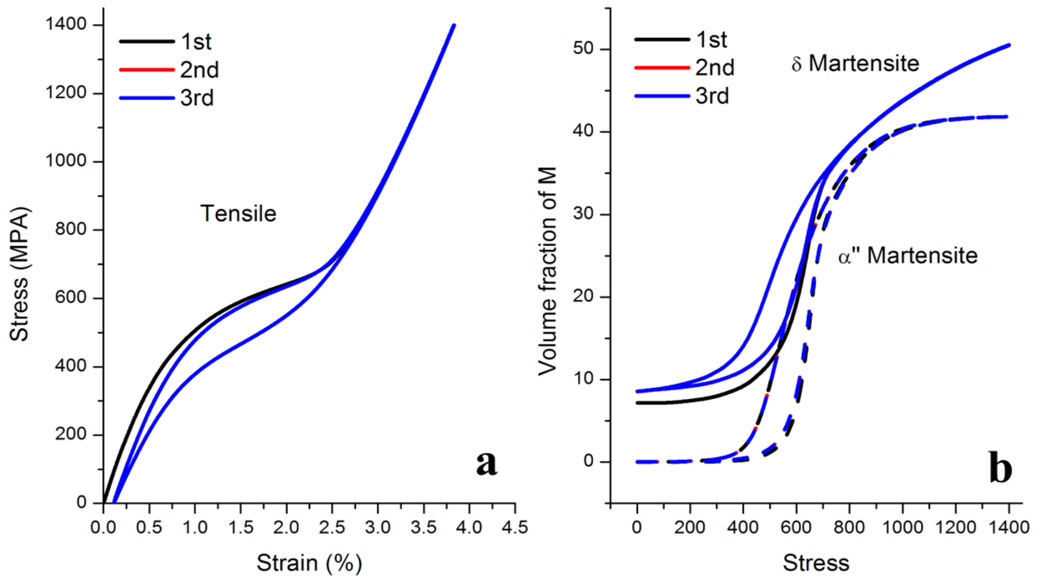

Figure S9 Phase field simulations on (a) the S-S curve for gum-type Ti-24Nb-4Zr-8Sn-0.10O single crystal loaded and unloaded along [110] orientation under different tension cycles and (b) related martensitic volume fraction in disordered B2 regime and ordered BCC regime under different loading cycles.

On-line video:

Movie S1 Evolution of HE-XRDS patterns with the X-ray incident beam along the $[001]_{\beta}$ for the Ti2448 single crystal during compressive loading along the $[110]_{\beta}$.

Movie S2 Evolution of HE-XRDS patterns with the X-ray incident beam along the $[001]_{\beta}$ for the Ti2448 single crystal during tensile loading along the $[110]_{\beta}$.

Movie S3 Evolution of HE-XRDS patterns with the X-ray incident beam along the $[1\bar{1}0]_{\beta}$ for the Ti2448 single crystal during tensile loading along the $[110]_{\beta}$.

References

1. Wang, D., Wang, Y., Zhang, Z. & Ren, X. Modeling Abnormal Strain States in Ferroelastic Systems: The Role of Point Defects. *Phys. Rev. Lett.* **105**, 205702 (2010).
2. Semenovskaya, S. & Khachaturyan, A.G. Coherent structural transformations in random crystalline systems. *Acta Mater.* **45**, 4367-4384 (1997).
3. Rao, W.F., Wuttig, M., & Khachaturyan, A.G. Giant Nonhysteretic responses of two-phase nanostructured alloys. *Physical Review Letters* **106** (10) (2011).
4. Morris, J. W., Jr. *et al.* Anomalous transformation-induced deformation in ⟨110⟩ textured Gum Metal. *Acta Mater.* **58**, 3271-3280 (2010).
5. Khachaturyan, A. G. Theory of Structural Transformations in Solids (John Wiley & Sons, New York, 1983).
6. Fischer-Cripps, A. C. & Lawn, B. R. Indentation stress-strain curves for “quasi-ductile” ceramics. *Acta Mater.* **44**, 519-527 (1996).
7. Zhang, Y. W. *et al.* Elastic properties of Ti–24Nb–4Zr–8Sn single crystals with bcc crystal structure. *Acta Mater.* **59**, 3081-3090 (2011).
